# A structural and biochemical comparison of Ribonuclease E homologues from pathogenic bacteria highlights species-specific properties

**DOI:** 10.1038/s41598-019-44385-y

**Published:** 2019-05-28

**Authors:** Charlotte E. Mardle, Thomas J. Shakespeare, Louise E. Butt, Layla R. Goddard, Darren M. Gowers, Helen S. Atkins, Helen A. Vincent, Anastasia J. Callaghan

**Affiliations:** 1 School of Biological Sciences and Institute of Biological and Biomedical Sciences, University of Portsmouth, Portsmouth, PO1 2DY United Kingdom; 2 Defence Science and Technology Laboratory, Porton Down, Salisbury, United Kingdom; 3 University of Exeter, Exeter, United Kingdom; 4 London School of Hygiene and Tropical Medicine, London, United Kingdom

**Keywords:** Enzymes, Nucleases, RNA decay, SAXS

## Abstract

Regulation of gene expression through processing and turnover of RNA is a key mechanism that allows bacteria to rapidly adapt to changing environmental conditions. Consequently, RNA degrading enzymes (ribonucleases; RNases) such as the endoribonuclease RNase E, frequently play critical roles in pathogenic bacterial virulence and are potential antibacterial targets. RNase E consists of a highly conserved catalytic domain and a variable non-catalytic domain that functions as the structural scaffold for the multienzyme degradosome complex. Despite conservation of the catalytic domain, a recent study identified differences in the response of RNase E homologues from different species to the same inhibitory compound(s). While RNase E from *Escherichia coli* has been well-characterised, far less is known about RNase E homologues from other bacterial species. In this study, we structurally and biochemically characterise the RNase E catalytic domains from four pathogenic bacteria: *Yersinia pestis*, *Francisella tularensis*, *Burkholderia pseudomallei* and *Acinetobacter baumannii*, with a view to exploiting RNase E as an antibacterial target. Bioinformatics, small-angle x-ray scattering and biochemical RNA cleavage assays reveal globally similar structural and catalytic properties. Surprisingly, subtle species-specific differences in both structure and substrate specificity were also identified that may be important for the development of effective antibacterial drugs targeting RNase E.

## Introduction

For survival, microorganisms must have the ability to rapidly adapt to environmental changes. One mechanism for achieving this is regulation of gene expression through the differential processing and/or turnover of RNAs by ribonucleases (RNases)^1^. The importance of RNases in these processes is underlined by the roles that they play in pathogenic bacterial virulence^2^ and, as a consequence, RNases are emerging as attractive antibacterial drug targets^2–4^. For example, the endoribonuclease RNase E is essential for cell viability in *Escherichia coli*^5–7^ where roles in mRNA decay and the maturation of tRNA and rRNA have been well documented [reviewed in^1^ and^8^]. RNase E is also required for cell viability in *Salmonella enterica*^9^ and has been implicated in the pathogenicity of both *S. enterica* and *Yersinia pestis*^10,11^. Furthermore, since homologues of RNase E are predicted to be present in many bacteria^12^, including pathogenic species, but are not found in animals or humans, RNase E is an ideal potential antibacterial target^2–4,13^.

*E. coli* RNase E (*Ec*RNase E) is the founding member of Type I RNase Es, found in betaproteobacteria, gammaproteobacteria and cyanobacteria^12,14^, and has been extensively characterised. It is a large protein containing 1061 amino acids and can be divided into two domains. The N-terminal domain (NTD) is responsible for the endoribonuclease activity^15^ and the C-terminal domain (CTD) forms the structural scaffold for the RNA-degrading multienzyme complex, the degradosome^16,17^. The catalytic NTD consists of five subdomains: an RNase H domain, an S1 domain, a 5′ sensor, a deoxyribonuclease (DNase) I domain and a small domain^18^ (Supplementary Fig. S1). It is a homotetramer formed by interactions between small domains^18–21^. The catalytic site is located in the DNase I domain and harbours a hydrated magnesium ion, coordinated by two aspartic acids, Asp_303_, positioned by asparagine Asn_305_, and Asp_346_, that is essential for the hydrolytic cleavage of the RNA substrate^18^. *Ec*RNase E cleaves single-stranded A/U-rich regions^22^ and has a strong preference for a 5′ monophosphate^23^. Specificity for a uracil at the +2 position relative to the cleavage site is defined by the uracil pocket in the S1 domain^24^. This pocket is comprised of Phe_67_, positioned by Phe_57_, and the Lys_112_-Gly_113_-Ala_114_-Ala_115_ (KGAA) loop^24^. In addition, recognition of a 5′ monophosphorylated substrate requires the 5′ sensor pocket, formed by amino acids Gly_124_, Val_128_, Arg_141_, Arg_142_, Arg_169_, Thr_170_ and Arg_373_ with Val_128_, Arg_169_ and Thr_170_ playing the critical roles in 5′ monophosphate detection^18,25^. Binding of the RNA substrate by the 5′ sensor is predicted to induce a significant structural conformational change that helps to correctly position the RNA substrate for cleavage^26^.

A number of RNase E homologues from other species have been partially characterised [reviewed in^12^]. In general, the sequence of the catalytic region of RNase E is highly conserved, whereas the sequence of the degradosome scaffold region is more variable^12^. Consequently, the focus has mostly been on the degradosome scaffold and its interacting partners^12^. However, there are indications that the properties of the catalytic domain are species-specific^27^ and that species-specific differences can extend to the response to potential antibacterial compounds shown to inhibit *Ec*RNase E^13^. Therefore, characterising the properties of the RNase E catalytic domain from a variety of species may be critical for the development of effective antibacterial drugs targeting RNase E. In this study, we have characterised the structural and biochemical properties of the catalytic domain of the RNase E homologue from four bacterial pathogens, chosen because of their importance in both the health and defence sectors. Specifically, RNase E homologues were chosen from bubonic plague causing *Y. pestis* (*Yp*RNase E), tularemia causing *Francisella tularensis* (*Ft*RNase E), melioidosis causing *Burkholderia pseudomallei* (*Bp*RNase E) and ESKAPE pathogen^28^ *Acinetobacter baumannii* (*Ab*RNase E). The sequence and structure of the RNase E catalytic domains were initially investigated *in silico*, before comparing their low-resolution solution structures to that of *Ec*RNase E by small-angle x-ray scattering (SAXS). The substrate specificity was also compared to that of *Ec*RNase E using biochemical assays. Collectively, these studies have revealed subtle species-specific differences in the properties of previously uncharacterised RNase E catalytic domains from pathogenic species that may prove to be important for the development of effective antibacterial compounds targeting RNase E.

## Results

### Sequence analysis of RNase E NTDs

**Figure 1:**
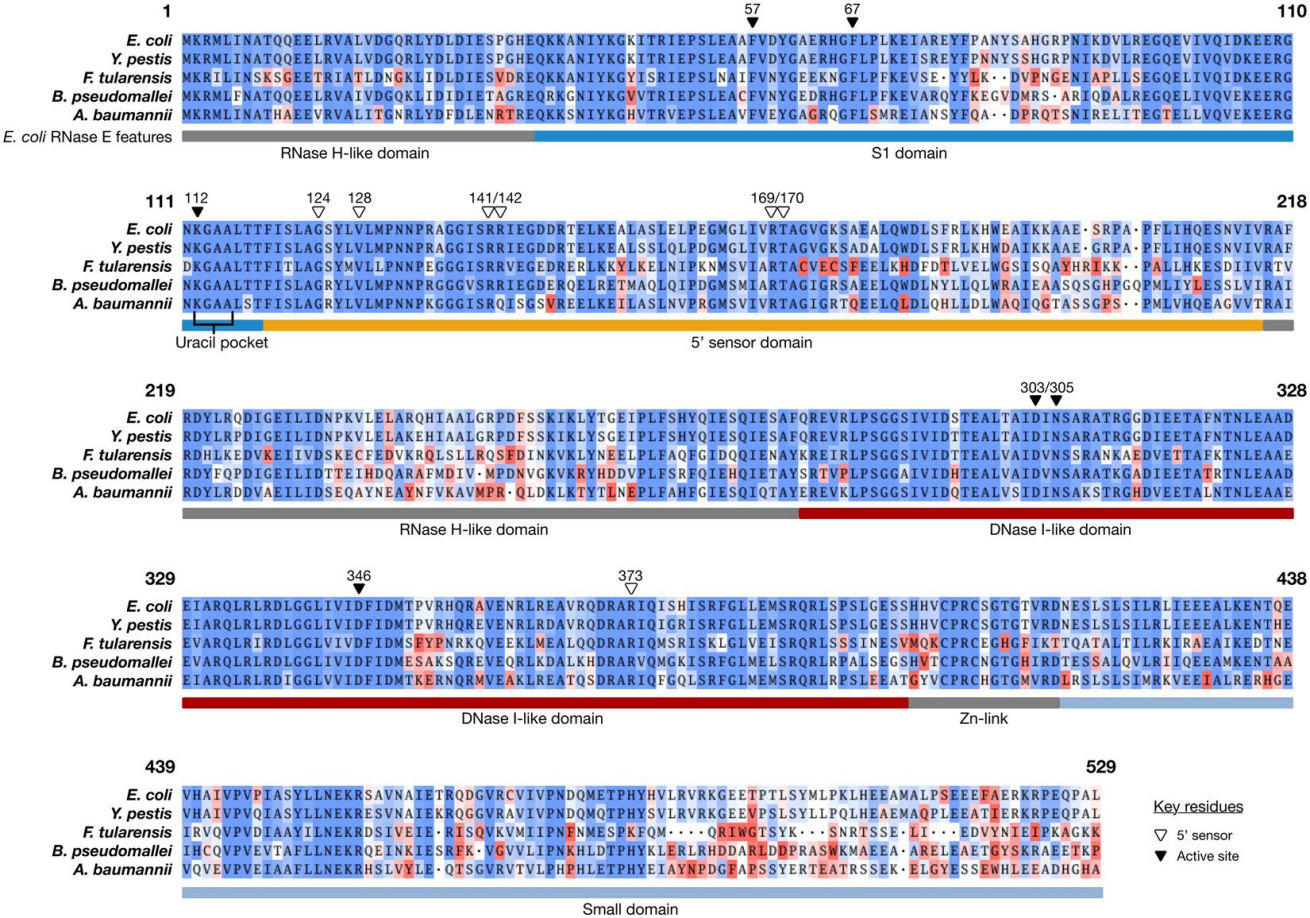
Multiple protein sequence alignment of RNase E catalytic domains. A multiple protein sequence alignment of RNase E homologues from *E. coli*, *Y. pestis*., *F. tularensis*, *B. pseudomallei* and *A. baumannii*. The region corresponding to the catalytic domain is shown and is coloured in accordance with amino acid side chain using a heatmap based on a BLOSUM62 matrix. Coloured bars below the alignment indicate the subdomains within the catalytic domain of *E. coli* RNase E (see Supplementary Fig. S1 for a structural representation). The alignment numbering is based on *E. coli* RNase E. Key amino acids forming the 5′ sensor pocket and active site^18,25^ are denoted with white and black triangles, respectively.

In order to compare the protein sequences of the RNase E homologues from the gammaproteobacteria *E. coli*, *Y. pestis*, *F. tularensis*, and *A. baumannii* and betaproteobacteria *B. pseudomallei* a multiple sequence alignment of the five full-length proteins was generated and trimmed to the boundaries of the *Ec*RNase E NTD (Fig. 1). As expected, based on their phylogeny, *Yp*RNase, *Ft*RNase E, *Ab*RNase and *Bp*RNase E all belong to the Type I class of RNase Es with catalytic domains of a similar length to *Ec*RNase E NTD located at the N-terminus of the protein. Consequently, from this point forwards, we will also refer to these catalytic domains as NTDs. The alignment reveals that the amino acid sequence of the NTD is highly conserved, with similarities of 69.4% for *Ft*RNase E, 70.5% for *Ab*RNase E, 75.4% for *Bp*RNase E and 96% for *Yp*RNase E, relative to *Ec*RNase. Furthermore, for these five sequences, there is complete conservation of the key residues known to be critical for the specific recognition of substrates containing a 5′ monophosphate by the 5′ sensor pocket (Val_128_, Arg_169_ and Thr_170_, using numbering for *Ec*RNase E;^18,25^) and for substrate cleavage at the active site (Asp_303_, Asn_305_ and Asp_346_, using numbering for *Ec*RNase E;^18^) (Fig. 1).

### *In silico* structural analysis of the RNase E NTDs

**Figure 2:**
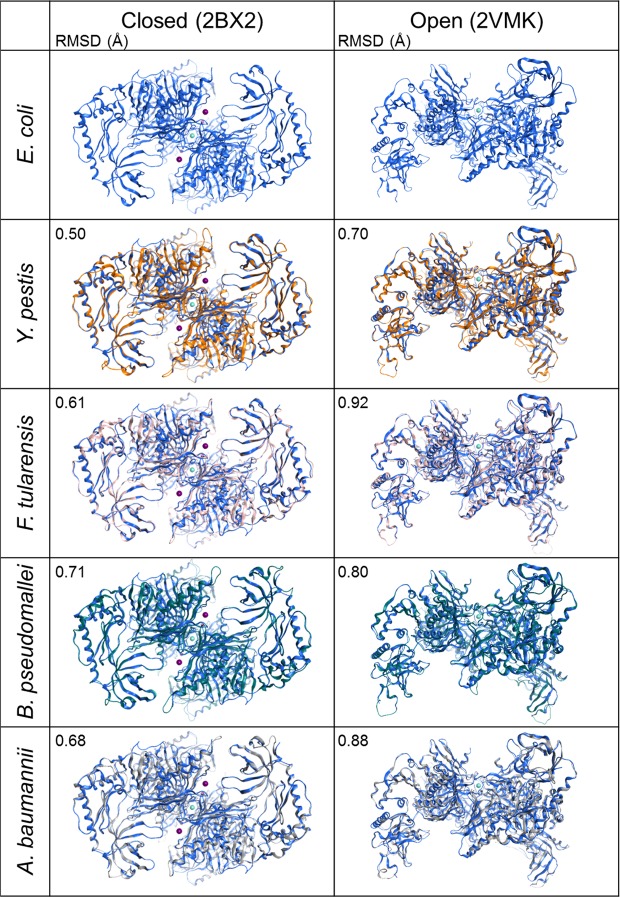
Homology models of RNase E NTDs. Crystal structures of the closed (2BX2)^18^ and open (2VMK)^26^ conformations of *E. coli* RNase E NTD (blue ribbons) and homology models of *Y. pestis*, *F. tularensis*, *B. pseudomallei* and *A. baumannii* RNase E NTDs (various coloured ribbons). The Mg^2+^ ions are shown as magenta spheres and the Zn^2+^ ions are shown as cyan spheres. The averaged homology models have been overlaid with the *E. coli* RNase E NTD crystal structure that was used as the template. The RMSD for each homology model in comparison with the template structure is reported in the top left-hand corner of the homology model panel.

In order to begin to investigate whether the amino acid sequence similarity also correlates with structural similarity, we decided to generate homology models for *Yp*RNase E, *Ft*RNase E, *Bp*RNase E and *Ab*RNase E NTDs using available *Ec*RNase E NTD crystal structures as templates. The crystal structure of *Ec*RNase E NTD has been solved in the presence^18,25^ and absence^26^ of RNA substrates and collectively these structures suggest a large conformational change between an open and closed conformation upon substrate binding^26^. Both open and closed conformation homology models were generated using the *Ec*RNase E NTD crystal structures 2VMK^26^ and 2BX2^18^ as the template structure, respectively (Fig. 2). Globally, the homology models are very similar to the *Ec*RNase E NTD crystal structures and this is reflected in the low root-mean-square deviation (RMSD) obtained for each model compared to the template crystal structure (Fig. 2). Even regions that are poorly conserved at the sequence level (e.g. amino acids 175–203 of the 5′ sensor, 233–263 of the RNase H domain and 457–508 of the small domain (Fig. 1)) are predicted to adopt a similar conformation (Fig. 2; Supplementary Fig. 2). The RMSDs for the homology models generated using the closed *Ec*RNase E NTD crystal structure 2BX2^18^ as a template are slightly lower than those generated using the open *Ec*RNase E NTD structure 2VMK^26^ as the template. However, this most likely reflects the higher resolution of the closed structure (2.85 Å)^18^ compared to the open structure (3.3 Å)^26^. Overall, these data suggest that *Yp*RNase E, *Ft*RNase E, *Bp*RNase E and *Ab*RNase E NTDs are likely to fold into a similar structure and adopt similar conformations to *Ec*RNase E NTD. This also implies that the conformational change from open to closed proposed for *Ec*RNase E^26^ is, at least theoretically, possible.

### Purification of recombinant RNase E NTDs

In order to structurally and functionally characterise *Yp*RNase E, *Ft*RNase E, *Bp*RNase E and *Ab*RNase E NTDs, and compare their properties to those of *Ec*RNase E NTD, pure recombinant RNase E NTDs were produced. An expression plasmid that was previously used for the expression, purification and characterisation of an N-terminally His-tagged *Ec*RNase E NTD^19,29^ was obtained from Prof. Ben Luisi, University of Cambridge, UK. We adopted a similar cloning strategy for *Yp*RNase E, *Ft*RNase E, *Bp*RNase E and *Ab*RNase E NTDs. All five RNase E NTDs were expressed as N-terminally His-tagged proteins and purified by immobilised Ni^2+^-affinity chromatography, followed by size-exclusion chromatography, as described previously for *Ec*RNase E NTD^19^. All five RNase E NTDs were successfully purified using this approach (Supplementary Fig. S3).

### Low-resolution solution structure of RNase E NTDs

**Figure 3:**
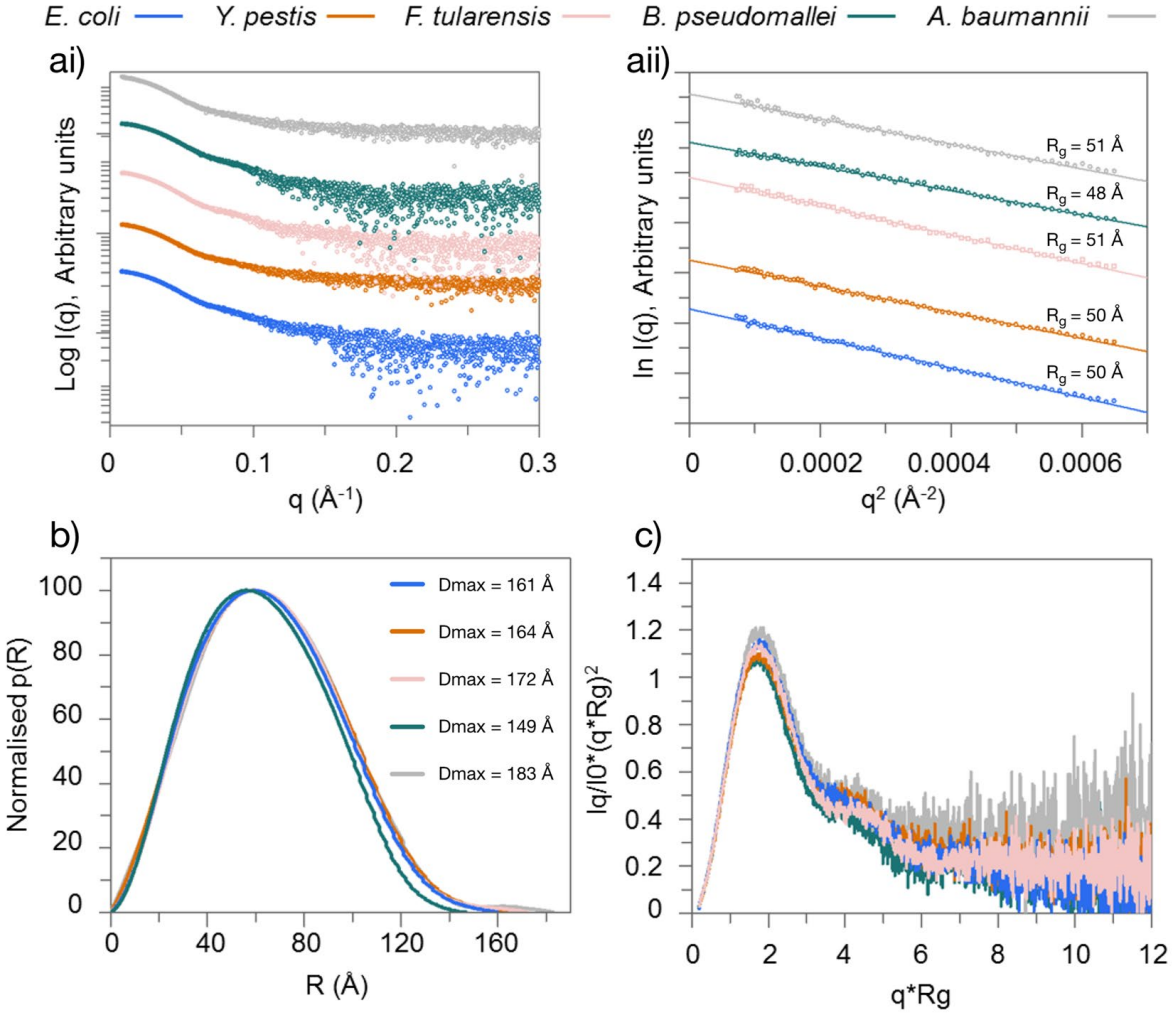
SAXS data and analysis for RNase E NTDs. (**ai**) Radially averaged scattering data for *E. coli*, *Y. pestis*., *F. tularensis*, *B. pseudomallei* and *A. baumannii* RNase E NTDs. Data have been plotted using an offset for visualisation purposes. (**aii**) Guinier region of the scattering data shown in ai for each of the RNase E NTDs together with fits and the derived R_g_ values. Data have been plotted using an offset for visualisation purposes. (**b**) Distance distribution p(R) plots for each of the RNase E NTDs together with the derived D_max_ values. Each plot has been normalised to the maximum p(R). (**c**) Dimensionless Kratky plots for each of the RNase E NTDs.

The catalytically active form of *Ec*RNase E NTD has been shown to be homotetrameric in solution^19^. During purification, *Yp*RNase E, *Ft*RNase E, *Bp*RNase E and *Ab*RNase E NTDs all eluted from the size-exclusion column at a similar volume to *Ec*RNase E NTD (Supplementary Table S1), which would be consistent with them also forming homotetramers. In order to further investigate, and compare, the structures of the RNase E NTDs, SAXS was employed. SAXS is a technique that is able to provide low resolution structural information about the size and shape of proteins in solution^30^. Parameters including molecular weight, radius of gyration (R_g_), which indicates the overall size of the protein, and maximum particle dimension (D_max_) can be derived from the scattering data, along with information about protein flexibility^30^. The scattering data for the RNase E NTDs are presented in Fig. 3a. Molecular masses were estimated from the SAXS profiles (Supplementary Table S1) and are consistent with each protein forming a tetramer in solution. For each RNase E NTD, the R_g_ was determined from Guinier analysis (Fig. 3aii). R_g_s ranged from 48 to 51 Å, indicating that all five RNase E NTDs are a similar overall size. The D_max_ was determined from the pair distance distribution (p(R)) plot (Fig. 3b), a plot of the distances between all possible pairs of atoms within the protien^30^, and ranged from 149 to 183 Å, suggesting some variability in the degree of extension of the proteins. Finally, dimensionless Kratky plots were used to assess the folding state and flexibility of the proteins^30^. The Kratky plots (Fig. 3c) were similar for each RNase E NTD and had the characteristic multiple bell-shaped profile of a multi-domain globular protein.

*Ab initio* models for *Ec*RNase E NTD were generated based on the SAXS data and were compared to three available *Ec*RNase E NTD crystal structures, 2BX2 (closed conformation)^18^, 2VMK (open conformation)^26^ and 5F6C (a transitioning conformation)^25^ (Supplementary Fig. S4a). None of the crystal structures could be fitted entirely within the average molecular envelope. The experimental scattering data for *Ec*RNase E NTD was also compared to theoretical scattering data for the three crystal structures (Supplementary Fig. S4b). We compared the resulting χ^2^ values to evaluate which of the crystallised conformations the solution scattering data most closely resembles. For *Ec*RNase E NTD, the data are most consistent with the transitioning conformation (Supplementary Table 2; Supplementary Fig. S4b). A similar comparison of the experimental scattering data for *Yp*RNase E, *Ft*RNase E, *Bp*RNase E and *Ab*RNase E NTDs to the theoretical scattering data for the three *Ec*RNase E crystal structures was also performed. This revealed that, in solution, *Yp*RNase E, *Ft*RNase E and *Ab*RNase E also most closely resemble the transitioning *Ec*RNase E NTD conformation (Supplementary Table S2). In contrast, *Bp*RNase E most closely resembles the closed *Ec*RNase E NTD conformation (Supplementary Table S2).

### Endoribonuclease Activity of RNase E NTDs

**Figure 4:**
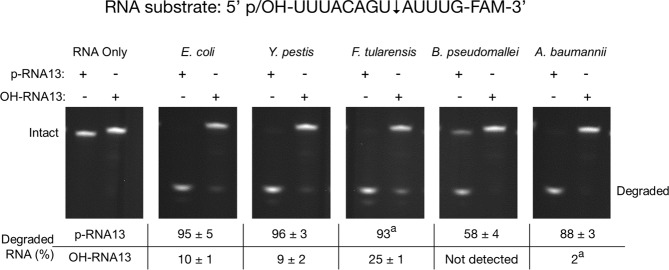
Cleavage of 5′-p-RNA13-FAM-3′ and 5′-OH-RNA13-FAM-3′ by RNase E NTDs. The sequence of the RNA substrates is shown, with an arrow indicating the expected cleavage site. 5 nM *E. coli*, *Y. pestis*, *F. tularensis*, *B. pseudomallei* or *A. baumannii* RNase E NTD were incubated with 1 μM 5′-p-RNA13-FAM-3′ (p-RNA13) or 1 μM 5′-OH-RNA13-FAM-3′ (OH-RNA13) at 28 °C for 45 minutes. Reaction products were resolved by 20% urea-PAGE and visualised using a G:Box UV transilluminator (Syngene). This figure is assembled from multiple gels that have been cropped. The uncropped gels are presented in Supplementary Fig. S5. The percentage of cleaved RNA present in each lane is indicated below the gels. Values are an average of three experiments, values have been rounded to the nearest whole numbers. Errors, where given, are the standard deviation to the nearest whole number (^a^calculated error was below 0.5).

Having established that *Ec*RNase E, *Yp*RNase E, *Ft*RNase E, *Bp*RNase E and *Ab*RNase E NTDs are all homotetramers that adopt similar global conformations in solution, we next wanted to investigate their catalytic properties. We decided to assess the endoribonuclease activity of each of the RNase E NTDs using a model 5′ p-RNA13-FAM-3′ substrate in a discontinuous end-point assay (Fig. 4). RNA13 has the same nucleotide sequence as the 13-mer oligoribonucleotide that was crystallised in complex with *Ec*RNase E NTD^18^ and is based on a known *Ec*RNase E cleavage site within RNAI from the pBR322 plasmid^31^. Endoribonucleolytic cleavage is expected to occur between the 8^th^ and 9^th^ nucleotide from the 5′ end of the substrate to generate an unlabelled octamer and a 3′ FAM-labelled pentamer (Fig. 4). Both the disappearance of the 3′ FAM-labelled 13-mer substrate and the appearance of the 3′ FAM-labelled pentamer product can be monitored by denaturing polyacrylamide gel electrophoresis (PAGE). As expected, *Ec*RNase E NTD efficiently cleaved this substrate such that 95% of the substrate had been degraded at the assay end-point (Fig. 4). *Yp*RNase E, *Ft*RNase E, *Bp*RNase E and *Ab*RNase E NTDs also endoribonucleolytically cleaved 5′ p-RNA13-FAM-3′ (Fig. 4). The cleavage efficiency for *Yp*RNase and *Ft*RNase E NTDs was similar to *Ec*RNase E NTD (96% and 93% of the substrate had been degraded at the assay end-point, respectively). However, *Bp*RNase E and *Ab*RNase E NTDs both cleaved 5′ p-RNA13-FAM-3′ less efficiently than *Ec*RNase E NTD (58% and 88% of the substrate had been degraded at the assay end-point, respectively).

### Substrate specificity of RNase E NTDs

*Ec*RNase E is known to have a strong preference for substrates with a 5′ monophosphate^23^ (e.g. 5′ p-RNA13-FAM-3′). In order to investigate whether *Yp*RNase E, *Ft*RNase E, *Bp*RNase E and *Ab*RNase E NTDs also share this preference we decided to compare the degradation of a 5′-OH-RNA13-FAM-3′, which has a 5′ hydroxyl, and 5′-p-RNA13-FAM-3′ in the discontinuous end-point assay (Fig. 4). As discussed above, *Ec*RNase E NTD efficiently cleaved the 5′-p-RNA13-FAM-3′ substrate with 95% substrate degraded at the assay end-point. However, as expected, cleavage of the 5′-OH-RNA13-FAM-3′ substrate was relatively inefficient and only 10% of this substrate was degraded by *Ec*RNase E NTD by the assay end-point. Similarly, compared to 5′ p-RNA13-FAM-3′, 5′-OH-RNA13-FAM-3′ was a poor substrate for *Yp*RNase E, *Ft*RNase E, *Bp*RNase E and *Ab*RNase E NTDs. However, while *Ec*RNase E, *Yp*RNase E, *Bp*RNase E and *Ab*RNase E NTDs degraded 10% or less of the 5′-OH-RNA13-FAM-3′ by the assay end-point, *Ft*RNase E NTD cleaved 25% of this substrate, 2.5-fold more than any of the other RNase E NTDs. Therefore, while all five RNase E NTDs display a preference for 5′ monophosphorylated substrates, this preference is less pronounced for *Ft*RNase E.

**Figure 5:**
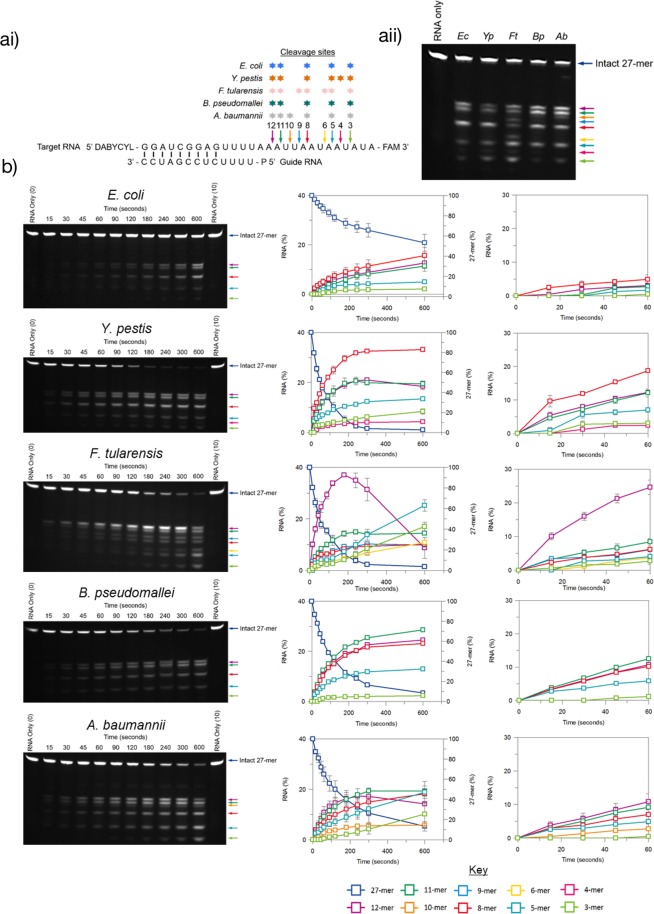
Cleavage site specificity of RNase E NTDs. (**ai**) Schematic of the target-guide RNA substrate. Coloured arrows indicate the position of cleavage sites that were observed for at least one of the RNase E NTDs, with the numbers above the arrows indicating the length of the 3′ FAM-labelled product that is produced by cleavage at that position. The cleavage sites that were observed for each homologue are indicated by stars above the respective position. (**aii**) 20% urea-PAGE analysis of cleavage of 1 μM target-guide substrate by 5 nM *E. coli*, *Y. pestis*, *F. tularensis*, *B. pseudomallei* or *A. baumannii* RNase E NTD after incubation at 28 °C for 10 minutes. The gel was visualised using a G:Box UV transilluminator (Syngene). The gel in this figure has been cropped and the uncropped gel is presented in Supplementary Fig. S6a. The band corresponding to the FAM-labelled 27-mer component of the target-guide substrate is labelled and the coloured arrows to the right of the gel correspond to the FAM-labelled cleavage products described in ai. (**b**) 20% urea-PAGE analysis of cleavage of 1 μM target-guide substrate by 5 nM *E. coli*, *Y. pestis*, *F. tularensis*, *B. pseudomallei* or *A. baumannii* RNase E NTD at 28 °C during a 10-minute time course. Gels were visualised using a G:Box UV transilluminator. The gels have been cropped and the uncropped gels are presented in Supplementary Fig. S6b. Coloured arrows to the right of the gels correspond to the cleavage positions depicted in ai. Graphs show the percentage of the FAM-labelled 27-mer component of the target-guide substrate and each of the FAM-labelled cleavage products at each time point for the entire time course (left) and the first minute of the time course (right). Data are the average of three experiments and error bars represent the standard deviation.

The short 5′-p-RNA13-FAM-3′ model substrate is cleaved by all of the RNase E NTDs at a specific, single site. However, longer, more complex substrates have the potential to be cleaved at multiple sites. For example, a partially double-stranded target-guide substrate consisting of a 5′ DABCYL-labelled, 3′ FAM-labelled 27-mer and a 5′ phosphorylated 13-mer with partial complementarity to the 5′ end of the 27-mer (Fig. 5ai) is known to be cleaved by *Ec*RNase E NTD at multiple sites, each with a different susceptibility to *Ec*RNase E^32^. Therefore, we decided to use the target-guide substrate in a discontinuous assay to compare cleave site specificity of the RNase E NTDs. In an initial experiment, 5 nM RNase E NTD was incubated with 1 μM target-guide RNA at 28 °C and the cleavage products were analysed by denaturing PAGE after 10 minutes (Fig. 5aii). *Ec*RNase E NTD cleaved the 27-mer component of the target-guide substrate at five positions: between nucleotides 15 and 16, 16 and 17, 19 and 20, 22 and 23 and 24 and 25, from the 5′ end, to generate 3′ FAM-labelled cleavage products 12, 11, eight, five and three nucleotides in length, respectively. Similar cleavage patterns were observed for *Yp*RNase E, *Bp*RNase E and *Ab*RNase E NTDs. Each of these RNase E NTDs also cleaved the 27-mer RNA at the positions cleaved by *Ec*RNase E NTD. Although additional cleavage sites were detected between nucleotides 17 and 18 for *Ab*RNase E NTD, generating a 3′ FAM-labelled product ten nucleotides in length, and between nucleotides 23 and 24 for *Yp*RNase E NTD, generating a 3′ FAM-labelled product four nucleotides in length. The relative predominance of the bands representing the common cleavage sites were also similar for *Ec*RNase E, *Yp*RNase E, *Bp*RNase E and *Ab*RNase E NTDs. In contrast, *Ft*RNase E NTD displayed a markedly different cleavage pattern to the other four RNase E NTDs. In addition to cleaving the 27-mer RNA at the five positions that are cleaved by the other RNase E NTDs, *Ft*RNase E NTD also cleaved the 27-mer RNA between nucleotides 18 and 19 and between nucleotides 21 and 22, generating 3′ FAM-labelled products nine and six nucleotides in length, respectively. Furthermore, the relative predominance of the bands representing the common cleavage sites was different for *Ft*RNase E NTD relative to the other four RNase E NTDs.

Given the observed similarities and differences in the cleavage patterns of the RNase E NTDs, time-course experiments were then used to identify initial cleavage sites and/or preferred cleavage sites (Fig. 5b). For *Ec*RNase E NTD, all five cleavage products began to appear from the start of the time-course, consistent with a distribution of single cleavage events. The octamer accumulated at the fastest rate, closely followed by the 12- and 11-mers suggesting that the preferred cleavage site is between nucleotides 19 and 20, from the 5′ end of the 27-mer. A similar accumulation of products was observed for *Yp*RNase E, *Bp*RNase E and *Ab*RNase E NTDs except that the 11-mer and the 12-mer accumulated at the fastest rate for *Bp*RNase E and *Ab*RNase E NTDs, respectively. For *Ft*RNase E NTD, the 12-mer accumulated significantly faster than the other cleavage products indicating that the preferred cleavage site is between nucleotides 15 and 16. Interestingly, after three minutes, the abundance of this cleavage product began to decline while the abundance of the trimer, pentamer and hexamer dramatically increased. This suggests that the initial 12-mer product can undergo a secondary cleavage to produce the shorter trimers, pentamers and hexamers.

## Discussion

We have investigated the structural and biochemical properties of the NTD of four, previously uncharacterised, RNase E homologues from bacterial pathogens and compared them to those of the well-characterised NTD from *E. coli* RNase E. Fundamentally, these NTDs are highly conserved at the sequence level and, as might have been expected, they have similar properties in that they are all homotetrameric, endoribonucleases. However, there are also subtle differences between them. The NTD from RNase E from *B. pseudomallei* is predicted to adopt a more closed conformation than the other RNase E NTDs (Fig. 3 and Supplementary Table S2) while the substrate specificity of *F. tularensis* RNase E NTD differs from the other RNase E NTDs (Figs 4 and 5).

Three principle regions of sequence variability were identified in the RNase E NTDs, amino acids 175–203, 233–263 and 457–508 (Fig. 1). Despite this sequence variability, the structure of these regions is predicted to be conserved (Figs 2 and S2). Two of these regions, one in the 5′ sensor domain (amino acids 175–203) and one in the RNase H domain (amino acids 233–263), are located at the peripheral edges of the tetrameric protein structure (Supplementary Fig. S2). These regions have no known direct catalytic function; however, given their location, it is possible that they may play a structural role in the movement of the 5′ sensor and S1 domains during the transition from an open to a closed conformation upon substrate binding. Interestingly, in solution, the RNase E NTD from *B. pseudomallei* adopts a more closed conformation than the other four RNase E NTDs (Fig. 3 and Supplementary Table S2) which could be related to the divergent sequence in this region. The third region of sequence variability, in the small domain (amino acids 457–508), is located at the protomer-promoter interface (Supplementary Fig. S2), where amino acid substitutions could affect tetramerisation. However, all five RNase E NTDs appear to be homotetrameric in solution (Supplementary Table S1), suggesting that the function of the small domain is conserved.

All of the RNase E NTDs investigated endoribonucleolytically cleaved model RNA substrates (Figs 4 and 5). This was not surprising given that the key residues known to be involved in RNA cleavage at the active site are absolutely conserved (Fig. 1). This study utilised model RNA substrates that we would expect to be cleaved via a 5′ end-dependent mechanism in which a 5′ monophosphate is specifically recognised by the 5′ sensor pocket^18,23,31^. The key residues known to be required for discriminating substrates with a 5′ monophosphate within this pocket are absolutely conserved in the RNase E NTDs investigated and, not surprisingly, all of the RNase E NTDs showed a preference for a 5′ monophosphorylated substrate (Fig. 4). However, more complex natural substrates such as 9S RNA, the precursor of 5S rRNA, can also be cleaved through an internal entry mechanism which relies on the recognition of a stem loop structure upstream of the cleavage site^33–35^. A recent study identified eight amino acids (Arg_3_, Gln_22_, His_268_, Tyr_269_, Gln_270_, Lys_433_, Arg_488_ and Arg_490_) in the *E. coli* RNase E NTD domain that are important for recognition of the stem loop^25^. These residues are absolutely conserved in *Y. pestis* RNase E but there are substitutions at two of the positions in *B. pseudomallei* and at six of the positions in *F. tularensis* and *A. baumannii* RNase Es. It remains to be seen whether these substitutions affect RNA cleavage through the internal entry mechanism for these RNase Es. Finally, substrate cleavage patterns varied between the RNase E NTDs, with the most striking differences observed for *F. tularensis* RNase E (Fig. 5), hinting at possible differences in the reaction mechanism.

In conclusion, we have characterised the RNase E NTD homologues from *Y. pestis*, *F. tularensis*, *A. baumannii* and *B. pseudomallei*. This is the first demonstration that these RNase E NTDs have endoriboucleolytic activity. Furthermore, through a comparison with *Ec*RNase E NTD, we have shown that, despite the expected global structural and catalytic similarities, there are also subtle species-specific differences between them. These observed subtle differences would not have been predicted *de novo* from amino acid sequence. The most significant differences were identified in two of the RNase E NTDs with the lowest sequence similarity, *B. pseudomallei* and *F. tularensis*. However, *B. pseudomallei* RNase E NTD varied in its structural properties while *F. tularensis* RNase E NTD varied in its biochemical properties. It remains to be determined, how significant these differences prove to be, but it does suggest caution when applying generalities based on studies with model organisms. In terms of the development of antibacterial drugs targeting RNase E, it has already been reported that RNase Es from different species respond differently to compounds selected based on inhibition of RNase E from *E. coli*^13^. Therefore, these subtle differences may prove to be critical when designing antibacterial approaches targeting RNase E in pathogens.

## Methods

### Protein sequence alignment

RNase E amino acid sequences were downloaded from UniProtKB using the following accession codes: *Ec*RNase E (P21513), *Yp*RNase E (Q74TC3), *Ft*RNase E (Q5NFK7), *Bp*RNase E (A0A0H3HN63) and *Ab*RNase E (A0A0B9WR03). The sequences were aligned in MOE (Molecular Operating Environment, 2013.08; Chemical Computing Group Inc., 1010 Sherbrooke St. West, Suite #910, Montreal, QC, Canada, H3A 2R7) using the default parameters, including a BLOSUM62 substitution matrix. This alignment was used to define the catalytic NTD for each of the RNase Es as the amino acids that aligned with amino acids 1–529 of *Ec*RNase E. Sequences were then trimmed to the NTD boundaries and the similarity of each homologue to *Ec*RNase E NTD was calculated in MOE.

### RNase E NTD homology models

RNase E NTD homology models were generated for *Yp*RNase E, *Ft*RNase E, *Bp*RNase E and *Ab*RNase E in MOE using the sequences that had been trimmed to the NTD boundaries (Fig. 1), *Ec*RNase E NTD crystal structures as the template structure (2BX2^18^ for the closed conformation and 2VMK^26^ for the open conformation) and the default settings. Ten initial modes were produced and averaged to give the most energetically favourable model. The RMSD between each model and the template *Ec*RNase E NTD structure was calculated in MOE.

### Cloning of RNase E NTD expression strains

RNase E NTDs were cloned into the pET16b expression plasmid (Novagen), in order to express them as N-terminally His_10_-tagged proteins. *Ec*RNase E NTD (aa 1–529) cloned into pET16b, was kindly provided by Prof. Ben Luisi (University of Cambridge, UK). The coding sequences for the RNase E NTDs were obtained from the European Nucleotide Archive: *Yp*RNase E (aa 1–529; CAL20235.1), *Ft*RNase E (aa 1–543; KFJ71366.1), *Bp*RNase E (aa 1–532; EET07304.1), and *Ab*RNase E (aa 1–544; A0A0B9WR03). The genes were codon-optimised *in silico* using GeneOtimizer® software (GeneArt, Life Technologies), synthesised by GeneArt and ligated between the *NdeI* and *BamHI* restriction sites of pET16b. All constructs were confirmed by DNA sequencing and *E. coli* BL21(DE3)pLysS was transformed with the plasmids for protein expression.

### Protein expression

RNase E NTD expression strains were grown in 500 ml LB supplemented with 100 μg/ml ampicillin, at 37 °C, with shaking (250 rpm), until the OD_600_ reached 0.6. Recombinant protein expression was induced by the addition of isopropyl β-D-1-thiogalactopyranoside (IPTG) to a final concentration of 1 mM and cells were incubated for a further 3 hours, at 37 °C, with shaking. Cells were harvested by centrifugation at 7,000 rcf and 4 °C for 20 minutes and stored at −20 °C.

### Protein purification

All RNase E NTDs were purified essentially as described previously for *Ec*RNase E NTD^19^. Briefly, frozen cell pellets were thawed on ice and resuspended in 50 ml of lysis buffer (20 mM Tris-HCl pH 8, 500 mM NaCl and 20 mM imidazole) supplemented with a cOmplete^TM^ ethylenediaminetetraacetic acid (EDTA)-free protease inhibitor cocktail tablet (Roche). Cells were lysed by sonication for 10 minutes (3.3 seconds on, 9.9 seconds off) using a Sonics Vibra Cell VCX 500 sonicator. Each lysate was clarified by centrifugation at 40,000 *g*, at 4 °C, for 20 minutes and was subsequently loaded onto a 5 ml HisTrap FF column (GE Healthcare) equilibrated in lysis buffer using an ÄKTA Purifier (GE Healthcare). Bound proteins were eluted with a linear gradient from 20 to 500 mM imidazole (0 to 100% elution buffer (20 mM Tris pH 8, 500 mM NaCl and 500 mM imidazole)) applied over six column-volumes. Fractions containing RNase E NTD were pooled and buffer-exchanged into storage buffer (20 mM Tris pH 8, 500 mM NaCl, 10 mM MgCl_2_, 10 mM EDTA, 10 mM dithiothreitol (DTT) and 10% (v/v) glycerol) using a HiPrep 26/10 Desalting column (GE Healthcare) and an ÄKTA Purifier. RNase E NTD was concentrated using a Vivaspin 20 centrifugal concentrator with a molecular weight cut-off (MWCO) of 10 kDa (Sartorius) and loaded onto a HiLoad 16/600 Superdex 200 prep grade size-exclusion column (GE Healthcare) equilibrated in storage buffer using an ÄKTA Purifier. Fractions containing RNase E were pooled, concentrated using a Vivaspin 20 (MWCO 10 kDa) centrifugal concentrator and stored at −80 °C. The size and homogeneity of the purified RNase E NTDs was assessed by 12% SDS-PAGE. The identity of the purified protein was confirmed by liquid chromatography tandem mass spectroscopy (LC-MS-MS; Astbury Centre, University of Leeds, UK).

### Size-exclusion chromatography coupled small-angle x-ray scattering (SEC-SAXS)

SEC-SAXS experiments were performed at the B21 beamline at the Diamond Light Source (DLS, Didcot, UK). RNase E NTDs were concentrated using a Vivaspin 20 (MWCO 10 kDa) centrifugal concentrator to a concentration of 5–10 mg/ml in sample buffer (10 mM DTT, 10 mM MgCl_2_, 0.5 M NaCl, 20 mM Tris (pH 8)). 45 μl were loaded onto a 4.6 ml KW403–4F size-exclusion column (Showdex) equilibrated in sample buffer at a flow rate of 0.1 ml/min, using an Agilent 1260 HPLC system. SAXS data were collected at 3-second intervals, at a wavelength of 12.4 KeV and a fixed camera length of 4.014 m using a Pilatus 2 M photon counting detector (Dectris), for 32 minutes. The data were normalised to the intensity of the incident beam and the scattering from the buffer was subtracted using an in-house program. Further data processing was then performed with the ATSAS suite^36^. PRIMUS^37^ was used to calculate the R_g_ and forward scattering intensity (I(0)) for the RNase E NTDs. I(0) was used to calculate the molecular weight (MW). The distance distribution function p(R) was generated with GNOM^38^ and was used to determine the D_max_. For *Ec*RNase E NTD 20 *ab initio* models were generated in DAMMIF^39^ and averaged in DAMAVER^40^. Crystal structures for *Ec*RNase E NTD in a closed conformation (2BX2;^18^), open conformation (2VMK;^26^) and a transitioning conformation (5F6C;^25^) were fitted to the averaged model using CHIMERA^41^. The theoretical scattering, based on the crystal structures, for *Ec*RNase E NTD in each of these three conformations was compared to the experimental scattering for each RNase E NTD in CRYSOL^42^.

### RNase E discontinuous assay

Discontinuous RNase E assays were carried out using one of three model RNA substrates: 5′ phosphorylated, 3′ FAM-labelled UUU ACA GUA UUU G (5′-p-RNA13-FAM-3′) 13-mer^18,31^; 5′ hydroxylated, 3′ FAM-labelled UUU ACA GUA UUU G (5′-OH-RNA13-FAM-3′) 13-mer or a partially double-stranded substrate (target-guide;^32^) generated by annealing a 5′ 4-(4-dimethylaminophenyl) diazenylbenzoic acid (DABCYL)-labelled, 3′ FAM-labelled GGA UCG GAG UUU UAA AUU AAU AAU AUA 27-mer and a 5′ phosphorylated UUU UCU CCG AUC C 13-mer at 28 °C for 15 minutes. The labelled 27-mer and unlabeled 13-mer were annealed at a ratio of 1:1.14 to ensure that all of the labelled 27-mer was partially double-stranded. All RNAs were obtained from Dharmacon. 30 μl reaction mixtures containing 1.25 to 5 nM RNase E NTD (see Figure legends for details), 1 μM substrate (see Figure legends for details), 25 mM Tris pH 8, 100 mM NaCl, 15 mM MgCl_2_, 1 mM DTT, 37.5 mg/ml Ficoll 70 and 5% (v/v) dimethyl sulfoxide (DMSO) were incubated at 28 °C for the indicated time(s). Reactions were terminated by the addition of 0.5 volumes of quench buffer (95% (v/v) formamide, 20 mM EDTA) and reaction products were resolved by denaturing 7.5 M urea 20% PAGE. Gels were visualised using a G:Box UV transilluminator (Syngene) and, where indicated, quantified by densitometry using ImageJ (Rasband WS, ImageJ, U. S. National Institutes of Health, Bethesda, Maryland, USA, http://imagej.nih.gov/ij/, 1997–2016).

## Supplementary information

Supplementary Information

## Data Availability

SAXS data have been deposited at SASBDB under Accession Codes SASDE52, SASDE62, SASDE72, SASDE82 and SASDE92.
