# Supplementary material for: A structural and biochemical comparison of Ribonuclease E homologues from pathogenic bacteria highlights species-specific properties
Source: Sci Rep. 2019 May 28;9:7952. doi: 10.1038/s41598-019-44385-y (PMC6538622; doi:10.1038/s41598-019-44385-y)
Supplement: Supplementary file 1 — Supplementary Information [file 41598_2019_44385_MOESM1_ESM.pdf]

Supplementary Information

A structural and biochemical comparison of Ribonuclease E homologues from pathogenic bacteria highlights species-specific properties

Charlotte E. Mardle[1], Thomas J. Shakespeare[1], Louise E. Butt[1], Layla R. Goddard[1], Darren M. Gowers[1], Helen S. Atkins[2,3,4], Helen A. Vincent[1]* and Anastasia J. Callaghan[1]*

[1] School of Biological Sciences and Institute of Biological and Biomedical Sciences, University of Portsmouth, Portsmouth, PO1 2DY, United Kingdom

[2] Defence Science and Technology Laboratory, Porton Down, Salisbury, United Kingdom

[3] University of Exeter, Exeter, United Kingdom

[4] London School of Hygiene and Tropical Medicine, London, United Kingdom

* To whom correspondence should be addressed. Tel: +44 (0)23 9284 2055; Fax: +44 (0)23 9284 2070; Email: Anastasia.Callaghan@port.ac.uk or Helen.Vincent@port.ac.uk

Supplementary Table S1. RNase E NTDs are homotetrameric in solution.

RNase E NTD	Theoretical molecular weight of homotetramer (kDa)	Elution volume from size-exclusion (ml)	SAXS-derived molecular weight (kDa)
E. coli	247	60.58	276
Y. pestis	248	60.52	276
F. tularensis	256	59.37	289
B. pseudomallei	250	60.36	257
A. baumannii	253	60.39	299

Supplementary Table S2. Comparison of the experimental scattering data for the RNase E NTDs to theoretical scattering data for available *Ec*RNase E NTD crystal structures. Underlined values indicate the model to which the data are most consistent.

	χ^2 for experimental data compared to theoretical data for *Ec*RNase E NTD crystal structures		
RNase E NTD	2BX2 (closed)	2VMK (open)	5F6C (transitioning)
E. coli	3.57	3.59	<u>2.14</u>
Y. pestis	7.02	6.63	<u>5.18</u>
F. tularensis	4.07	4.07	<u>2.16</u>
B. pseudomallei	<u>2.03</u>	2.27	3.97
A. baumannii	7.88	7.47	<u>6.45</u>

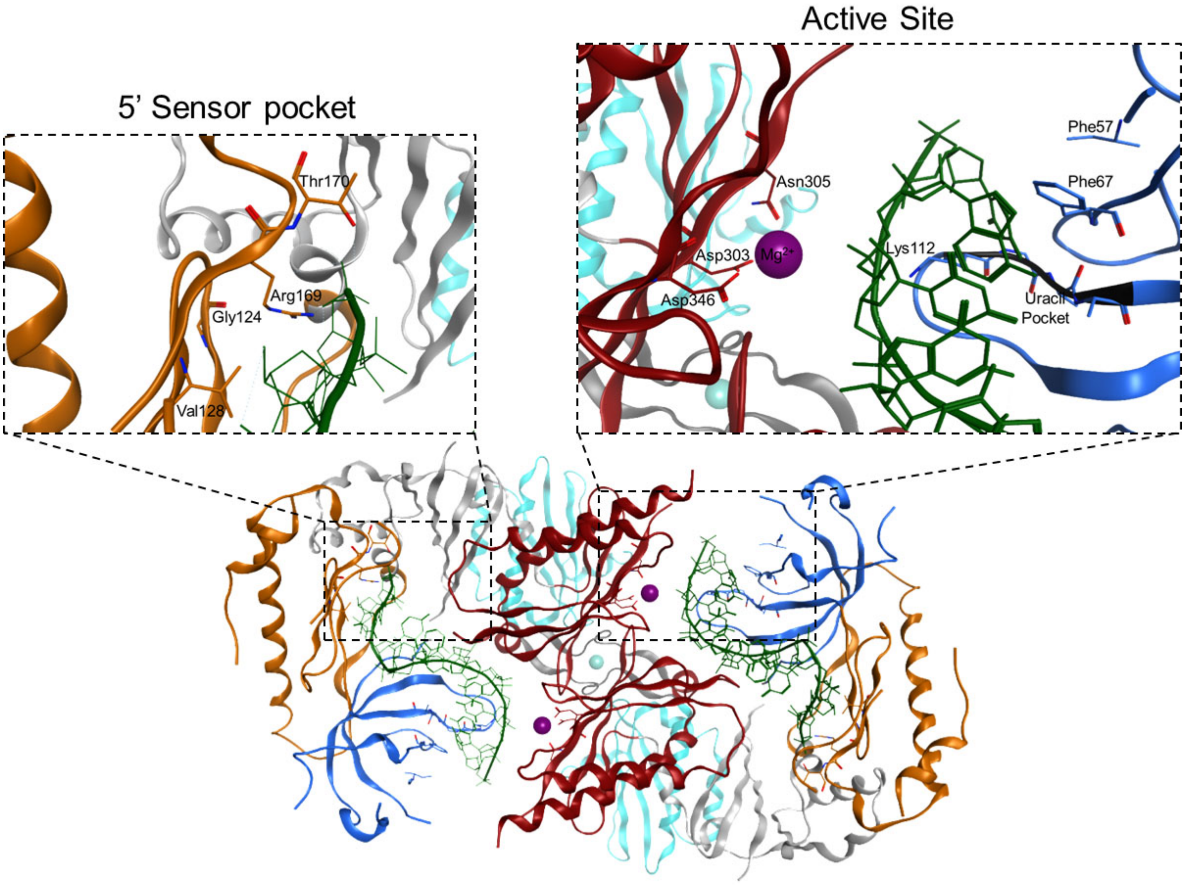

Supplementary Figure S1. Crystal Structure of *Ec*RNase E NTD. *Ec*RNase E dimer (PDB accession code: 2C0B; [18]) shown as ribbons and coloured by domain: RNase H domain and Zn-link (grey), S1 domain (blue) with the uracil pocket residues (KGAA loop; black)[24], 5’ sensor (gold), DNase I domain (red) and small domain (cyan). The Mg^{2+} ions are shown as magenta spheres, the Zn^{2+} ions as cyan spheres and the RNA substrate as green sticks. The 5’ sensor pocket and active site are enlarged in zoom-in panels with key amino acids, known to be essential for substrate recognition and/or cleavage, shown as labelled sticks[18,25].

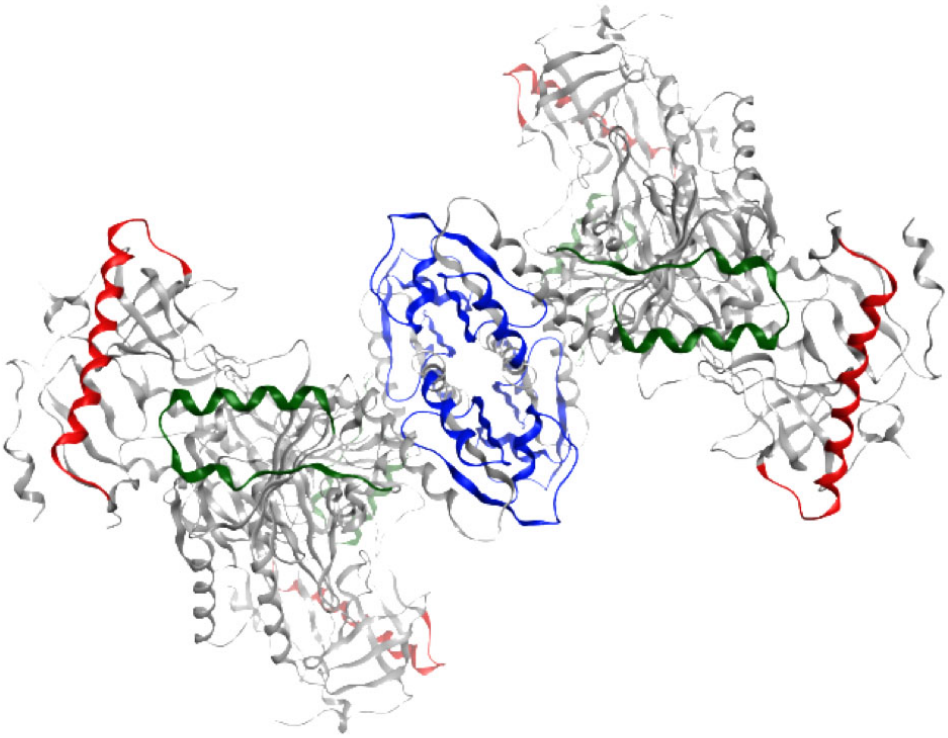

Supplementary Figure S2. Regions of protein sequence variability mapped onto the *Ec*RNase E NTD crystal structure. The *Ec*RNase E tetramer (PDB: 2BX2); [18]) is shown as grey ribbons with the poorly conserved regions (those corresponding to amino acids 175-203, 233-263 and 457-508 in the multiple sequence alignment in Fig. 1) coloured red (175-203), green (233-263) or blue (457-508), respectively.

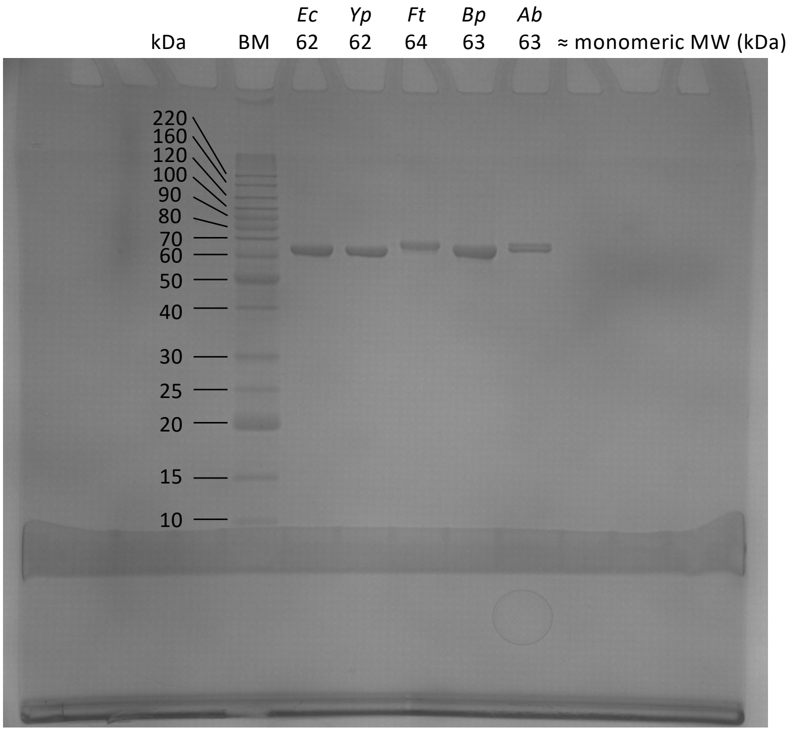

Supplementary Figure S3. RNase E NTD preparation. 12% SDS-PAGE of 1 μg purified *Ec*RNase E, *Yp*RNase E, *Ft*RNase E, *Bp*RNase E and *Ab*RNase E NTDs. BenchMark Protein Ladder (BM; Invitrogen) is provided as a molecular weight standard. The approximate theoretical molecular weight of the respective RNase E NTD monomer is indicated above the lane.

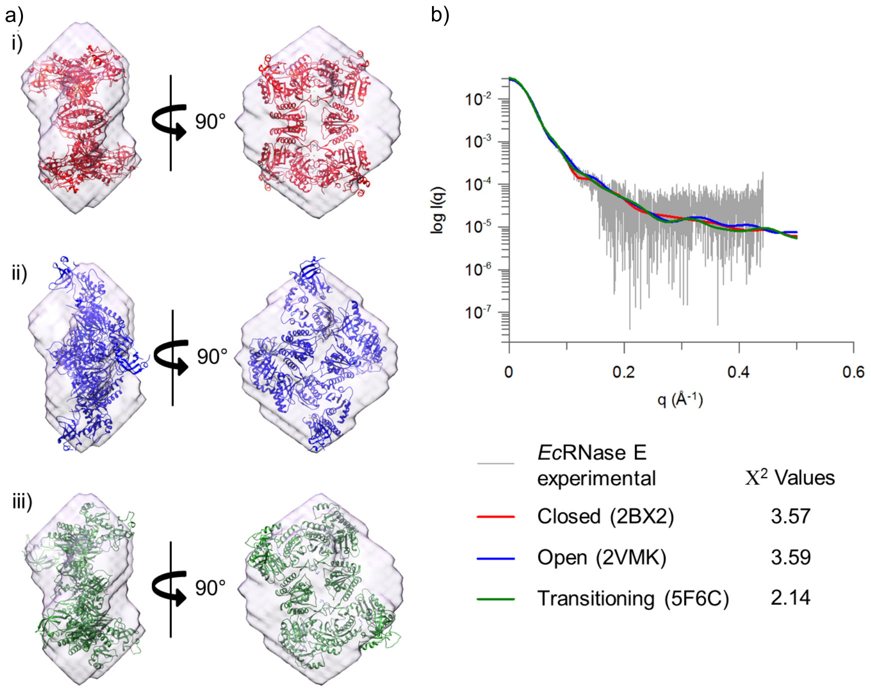

Supplementary Figure S4. Comparison of SAXS data for *E. coli* RNase E NTD to the available crystal structures. a) *E. coli* RNase E NTD crystal structures fitted into the average *ab initio* model. i) closed conformation *E. coli* RNase E crystal structure (PDB accession code: 2BX2; [18]), ii) open conformation *E. coli* RNase E crystal structure (PDB accession code: 2VMK; [26]) and iii) transitioning conformation *E. coli* RNase E crystal structure (PDB accession code: 5F6C; [25]). b) Theoretical scattering curves for *E. coli* RNase E NTD crystal structures and the experimental scattering data for *E. coli* RNase E NTD. χ^2 values for the theoretical scattering data compared to the experimental scattering data are shown.

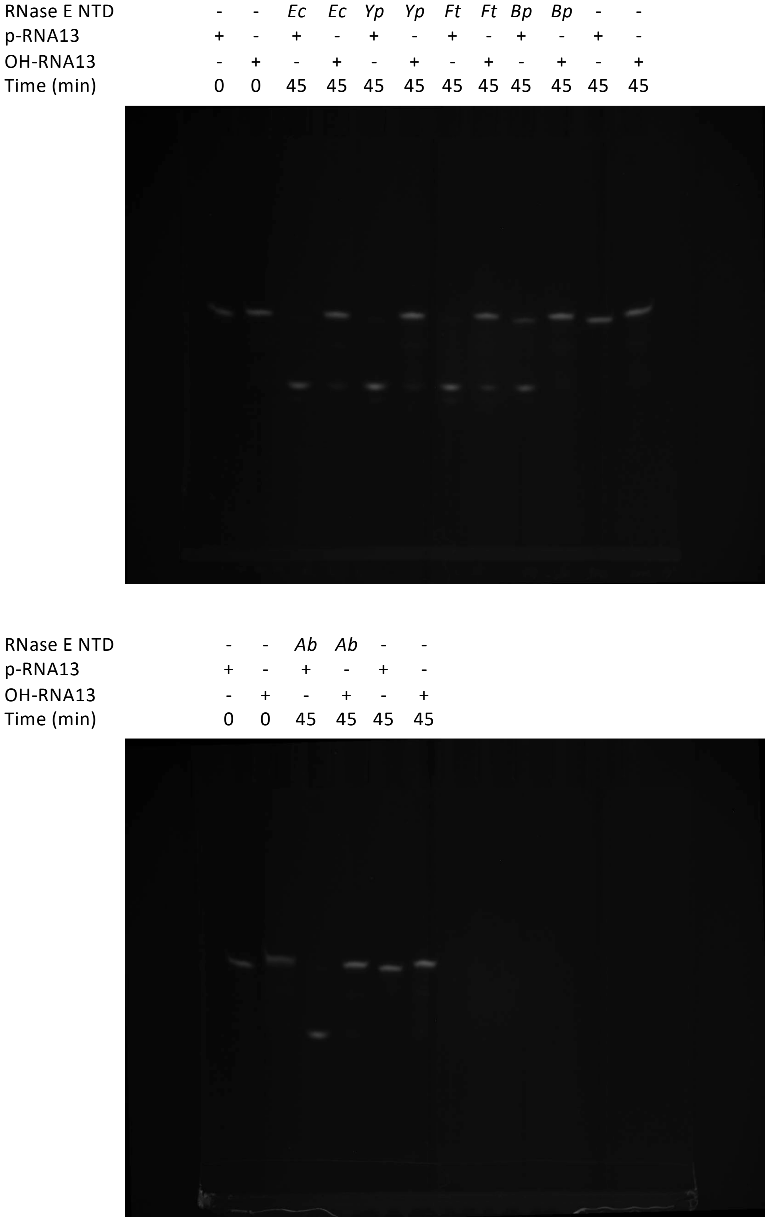

Supplementary Figure S5. Uncropped gel images that were used to prepare Fig. 4 (cleavage of 5′-p-RNA13-FAM-3′ and 5′-OH-RNA13-FAM-3′ by RNase E NTDs). 5 nM *E. coli*, *Y. pestis*, *F. tularensis*, *B. pseudomallei* or *A.baumannii* RNase E NTD were incubated with 1 μM 5′-p-RNA13-FAM-3′ (p-RNA13) or 1 μM 5′-OH-RNA13-FAM-3′ (OH-RNA13) at 28°C for 45 minutes. Reaction products were resolved by 20% urea-PAGE and visualised using a G:Box UV transilluminator (Syngene). To aid visualisation, contrast was adjusted to -20% for each gel in PowerPoint.

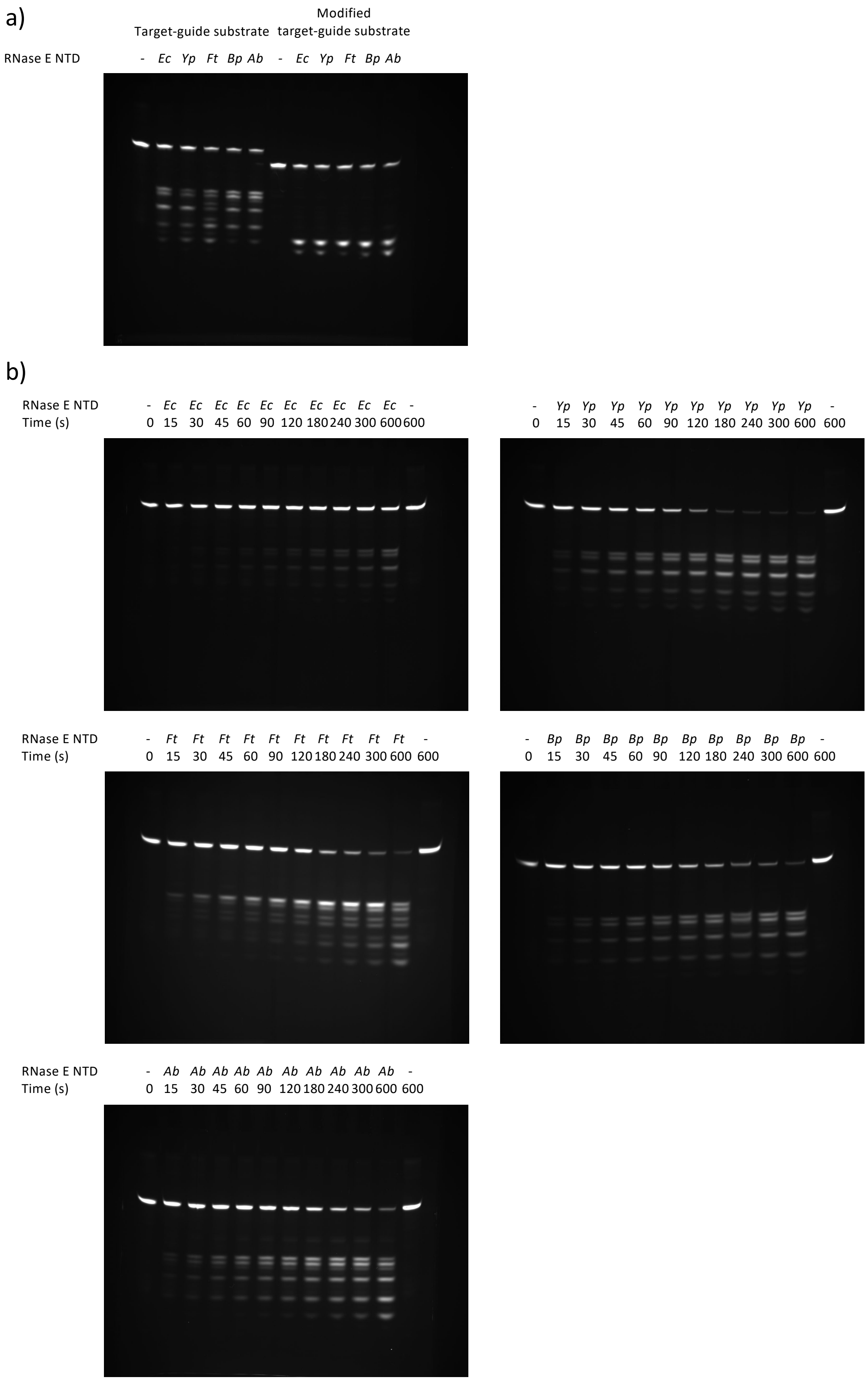

Supplementary Figure S6. Uncropped gel images that were used to prepare Fig. 5 (cleavage site specificity of RNase E NTDs). a) 20% urea-PAGE analysis of cleavage of 1 μM target-guide substrate (left six lanes) by 5 nM *E. coli*, *Y. pestis*, *F. tularensis*, *B. pseudomallei* or *A. baumannii* RNase E NTD

after incubation at 28°C for 10 minutes. The right six lanes of the gel are not relevant to this manuscript. The gel was visualised using a G:Box UV transilluminator (Syngene). b) 20% urea-PAGE analysis of cleavage of 1 μM target-guide substrate by 5 nM *E. coli*, *Y. pestis*, *F. tularensis*, *B. pseudomallei* or *A. baumannii* RNase E NTD at 28°C during a 10-minute time course. Gels were visualised using a G:Box UV transilluminator.
